# Hemostatic effect and safety evaluation of oxidized regenerated cellulose in total knee arthroplasty- a randomized controlledtrial

**DOI:** 10.1186/s12891-023-06932-7

**Published:** 2023-10-07

**Authors:** Bowei Li, Wenjie Pan, Xiaobo Sun, Kai Qin, Guoyang Bai, Huanli Bao, Yuanchi Huang, Jianbing Ma

**Affiliations:** 1 Department of Knee Joint Surgery, Honghui Hospital, Xi’an Jiaotong University, 555 East Friendship Road, South Gate, Xi’an, 710054 Shaanxi Province China; 2 Xi’an Medical University, Xi’an, 710068 Shaanxi Province China

**Keywords:** Oxidized regenerated cellulose, Total knee arthroplasty, Total blood loss, Hidden blood loss, Safety

## Abstract

**Background:**

Oxidized regenerated cellulose (ORC) is a type of biodegradable hemostatic material, which has been widely used in the field of surgery. However, its hemostatic effect in patients undergoing total knee arthroplasty (TKA) is uncertain. Accordingly, this study investigated the effectiveness and safety of ORC in patients receiving TKA.

**Methods:**

Seventy patients undergoing unilateral TKA were randomized into blank control group and ORC (2 pieces of ORC placed in the joint cavity) groups. Then, the two groups were compared for primary (perioperative blood loss [total blood loss, intraoperative blood loss, and hidden blood loss] and hemoglobin drop values) and secondary (coagulation indicators, inflammatory indicators,operation time, and complication rates) outcomes.

**Results:**

The total blood loss in the ORC group was 902.32 ± 307.82 mL, which was statistically significantly lower than that in the control group (1052.25 ± 308.44 mL) (*P* < 0.05). Postoperative hidden blood loss was also statistically markedly lower in the ORC group (801.61 ± 298.80 mL) than in the control group (949.96 ± 297.59 mL) (P < 0.05). There was no significant difference between the two groups in terms of coagulation indicators, inflammatory indicators, operation time, and complication rates.

**Conclusion:**

In conclusion, our prospective RCT study proved that regenerated oxidized cellulose can be used safely in vivo and can effectively reduce postoperative blood loss in patients, which is a potential method for preventing blood loss after TKA.

**Trial registration:**

This prospective RCT was reviewed and approved by the Ethics Committee of Honghui Hospital of Xi’an Jiaotong University (No: 202,211,007) and was designed and conducted according to the rules of the Declaration of Helsinki. Written informed consent was obtained from patients or their legal guardians.

## Background

Knee osteoarthritis (KNEE OA) is a chronic disorder characterized by articular cartilage degeneration, which frequently results in deformity and pain in the knee joint of patients. Patients with end-stage KNEE OA will ultimately pursue knee arthroplasty as the only viable option. Yet, TKA can also trigger significant blood loss, in addition to its advantage of greatly improving the pain and function of patients, and blood loss is attributed to the release of surrounding soft tissues, multiple intraoperative osteotomies and synovectomy [1, 2]. For patients with heavy blood loss, blood transfusion is often required. Nevertheless, blood transfusion elevates the risk of blood-borne diseases and immune disorders and may be associated with many complications, such as lung injury, renal failure, and coagulation disorders [3]. Overall, reducing post-TKA blood loss is critical to the postoperative rehabilitation of patients.

Hemostatic materials are extensively utilized clinically in orthopedic surgery and are effective for perioperative hemostasis. At present, the clinical hemostatic materials for orthopedic surgery are mainly categorized into 3 types: bone wax, hemostasis-osteogenesis integrated materials, and biodegradable materials [4].

Oxidized regenerated cellulose (ORC) is a type of biodegradable topical hemostatic material. In 1942, Yackel et al. first prepared oxidized cellulose and studied its property and characteristics [5]. According to their results, oxidized cellulose is derived from plants and has the advantages of safety, easy operation, and satisfactory efficacy in the clinic [6].Currently, ORC is widely used as an absorbable hemostatic material in various fields, including neurosurgery, thoracic surgery, general surgery, urology surgery, gynecology, etc. [7–10].However, orthopedic applications of ORC are currently limited, the hemostatic effectiveness of ORC during TKA and the effert of ORC indwelling in humans to reduce blood loss remains unclear. Most previous studies mainly indicated that ORC effectively reduced intraoperative blood loss, and seldom involved whether ORC indwelling in the human body can reduce blood loss. Therefore, the main goal of this randomized controlled trial is to detect whether ORC indwelling in humans can reduce perioperative blood loss, especially postoperative hidden blood loss, and its safety.

To this end, this prospective randomized control trial (RCT) was designed to analyze the effectiveness and safety of ORC in patients receiving TKA. We hypothesized that the use of ORC, a biodegradable local hemostatic material, is effective in diminishing perioperative blood loss, especially postoperative hidden blood loss, in patients receiving TKA, and also has a good safety profile.

## Methods

### Study design

This prospective RCT was reviewed and approved by the Ethics Committee of Honghui Hospital of Xi’an Jiaotong University (No: 202,211,007) and was designed and conducted according to the rules of the Declaration of Helsinki. Written informed consent was obtained from patients or their legal guardians.

### Power analysis

The sample size was calculated using a pilot study with 10 patients. For 1:1 parallel control, 28 patients were required in each group to obtain a statistical power of 0.90 and an α error of 0.05 for the two-sided test. Since 10% of patients might lose to follow-up and 10% might have incomplete data, this study recruited 35 patients per group, a total of 70 patients.

### Participants

Patients were consecutively screened according to inclusion and exclusion criteria and enrolled in this study. The inclusion criteria were patients undergoing initial unilateral TKA for end-stage osteoarthritis in this treatment team From December 1, 2022, to March 20, 2023, Exclusion criteria were as follows: patients with preoperative anemia (Hb concentrations < 12 g/dL in men and < 11 g/dL in women); patients with rheumatoid arthritis; patients with a history of arterial or venous thromboembolic diseases (such as deep vein thrombosis [DVT] or pulmonary embolism [PE]); patients with cerebrovascular diseases, patients with prior myocardial infarction, heart failure (New York Heart Association class III or IV), and atrial fibrillation; patients with hepatic and renal dysfunction; patients with genu varum, genu valgum, and flexion contracture greater than 30 degrees; patients with congenital or acquired coagulation disorders (preoperative international normalized ratio > 1.4, activated partial thromboplastin time > 1.4 × normal values, and platelets < 140,000/mm^3^); patients with intraoperative complications (such as fractures). In addition, patients who drop out or lose follow-up in the middle will also be excluded.

### Randomization and intervention

Participants (n = 70) were allocated into two groups by a blinded nurse with a computer-generated random number table in a double-blinded way: a control group, patients did not receive any local hemostatic measures during the surgery; an ORC group, patients were treated locally with ORC (Surgicel Fibrillar™ [2.5 * 5.1 cm]; Ethicon Inc., San Lorenzo, Puerto Rico, USA). The ratio of patients assigned to both groups was 1:1, with 35 patients in each group. The grouping of each patient was placed in an opaque sealed envelope by the blinded nurse and brought into the operating room prior to surgery. Prior to surgery, the envelope was opened by the operator to confirm the grouping of patients, followed by surgery. The person collecting the data did not participate in the study. The investigator analyzed the data after the entire experiment was completed.

### Surgery procedure

**Fig. 1:**
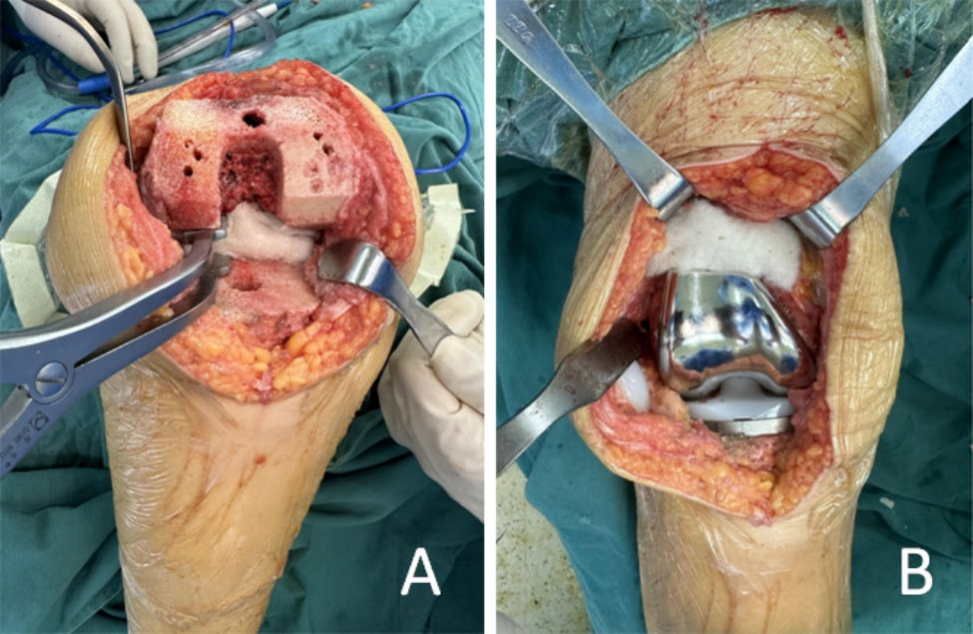
The procedure of intraoperative topical application of oxidized regenerated cellulose. **A**: Oxidized regenerated cellulose was placed on the posterior side of the joint capsule; **B**: Oxidized regenerated cellulose was placed on the suprapatellar bursa. Oxidized regenerated cellulose is a biodegradable hemostatic material

The surgeries for patients in both groups were performed by the same attending surgeon. Patients underwent general anesthesia and femoral nerve block(FNB) the affected extremity was routinely disinfected, followed by draping. Subsequently, a pneumatic tourniquet was applied. A 14-cm incision was intraoperatively created in the median skin of the knee joint, and the subcutaneous tissues and fascia were incised to remove hyperplasia, osteophytes, and synovial tissues in the joint. After osteotomy, a test mold was installed to check the balance of extension and flexion gap in the knee, and the prosthesis was adhered with bone cement and implanted. The same prosthesis (Beijing AK Medical Co., Ltd., Beijing, China) was used in both groups.Patients in the ORC group underwent hemostasis with two pieces of ORC, one piece on the posterior side of the articular capsule after the installation of the tibial prosthesis and the other piece on the suprapatellar bursa after the loosening of the tourniquet. Thereafter, the wound was sutured layer by layer (Fig. 1).Patients in the control group directly received wound sutures. No drainage tubes were used for all of the included cases.

### Postoperative care protocol

The wound area was iced every 3 h, 30 min/time, within 24 h after surgery. After 24 h, the patients were instructed to start out-of-bed activities, quadriceps strengthening exercises, and exercises for the range of motion of knee joints. All patients were given an intravenous drip of antibiotics (cefuroxime sodium; 1.5 g/100 mL) to prevent infection 6 and 24 h after surgery. Additionally, analgesia and thromboembolic prophylaxis were performed. Specifically, an analgesic pump was applied for postoperative self-administered analgesia. If patients were still in pain after the withdrawal of the pump, they were given oral analgesics (imrecoxib tablets, 0.1 g/time, until 2 months after discharge; paracetamol and dihydrocodeine tartrate tablets, 10 mg/time, from hospitalization to two weeks after surgery)twice daily for analgesia. Meanwhile, patients were subcutaneously injected with low-molecular-weight heparin calcium injection (4100IU in 0.4 mL, Hebei Changshan Biochemical Pharmaceutical Co., Ltd., Hebei, China; 0.4 mL/time) once daily during hospitalization until the third postoperative day and took rivaroxaban tablets (Qilu Pharmaceutical Co., Ltd., Shandong, China; 2.5 mg/day) once daily for 10 days after discharge. The routine blood test of patients was conducted on postoperative days 1 and 3. Blood transfusion was given when Hb concentrations were < 70 g/L, while blood transfusion was decided according to the physical compensation status of patients when Hb concentrations were between 70 and 100 g/L. None of the patients in this study received postoperative blood transfusion after evaluation. After discharge from the hospital, the patient continued to perform quadriceps strengthening exercises and knee joint range of motion exercises according to the doctor’s orders, and underwent regular reexamination until the knee joint function returned to normal.

### Outcomes

After admission, the baseline information of patients was obtained, such as gender, age, body mass index (BMI), American Society of Anesthesiologists (ASA) physical status classification, prothrombin time(PT), prothrombin time activity(PTA), international normalized ratio (INR), activatedpartial prothrombin time(APTT), thrombin time(TT), and fibrinogen(FIB).

The primary indicators were collected, including perioperative blood loss (total bloodloss, intraoperative bloodloss, and hidden blood loss) and Hb drop value.The total blood loss was calculated with the Gross method [11]:total blood loss = preoperative blood volume × (preoperative hematocrit [HCT] - postoperative HCT)/preoperative HCT. The preoperative blood volume(PBV) was calculated by referring to the method proposed by Nadler et al. [12]: preoperative blood volume = k1 × height^3^ (m) + k2 × body mass (kg) + k3 (for men: k1 = 0.3669, k2 = 0.03219, and k3 = 0.6041; for women: k1 = 0.3561, k2 = 0.03308, and k3 = 0.1833). The intraoperative blood loss was calculated with the following formula: intraoperative blood loss = (total fluid volume in the suction bottle + blood volume in the gauze - intraoperative flushing fluid volume), where blood volume in the gauze = postoperative gauze weight - net gauze weight. The hidden blood loss was calculated as follows: hidden blood loss = total blood loss - intraoperative blood loss. Hb drop values were calculated with the formula below: Hb drop value = preoperative Hb value - minimum Hb value during hospitalization.

Secondary indicators included the operation time, coagulation (D-dimer and fibrin degradation products [FDPs]) and inflammatory (C-reactive protein [CRP] and erythrocyte sedimentation rate [ESR]) indicators before surgery and on 1 and 3 days after surgery, and complications (blood transfusion, thrombosis, wound complications, early infection, and readmission) within 6 weeks.

Bilateral lower extremity venous ultrasound was performed for all patients on postoperative days 1 and 14. Patients were postoperatively followed up for 6 weeks, which included wound complications, infection, PE, myocardial infarction, and readmission.

### Statistical analysis

SPSS 25.0 statistical software was used to enter and process the data. Normally distributed data were expressed as mean ± standard deviation, and skewed data were expressed as median (interquartile range). Categorical variables (gender, ASA classification, and complications) were analyzed with Pearson’s chi-square testor continuity correction.

Continuous variables (age, BMI, Hb, HCT, platelets, PBV, blood loss, Hb drop value, operation time, CRP, ESR, D-dimer, and FDPs) were analyzed with the independent samples *t*-test. A difference was considered statistically significant at *P* < 0.05.

## Results

**Fig. 2:**
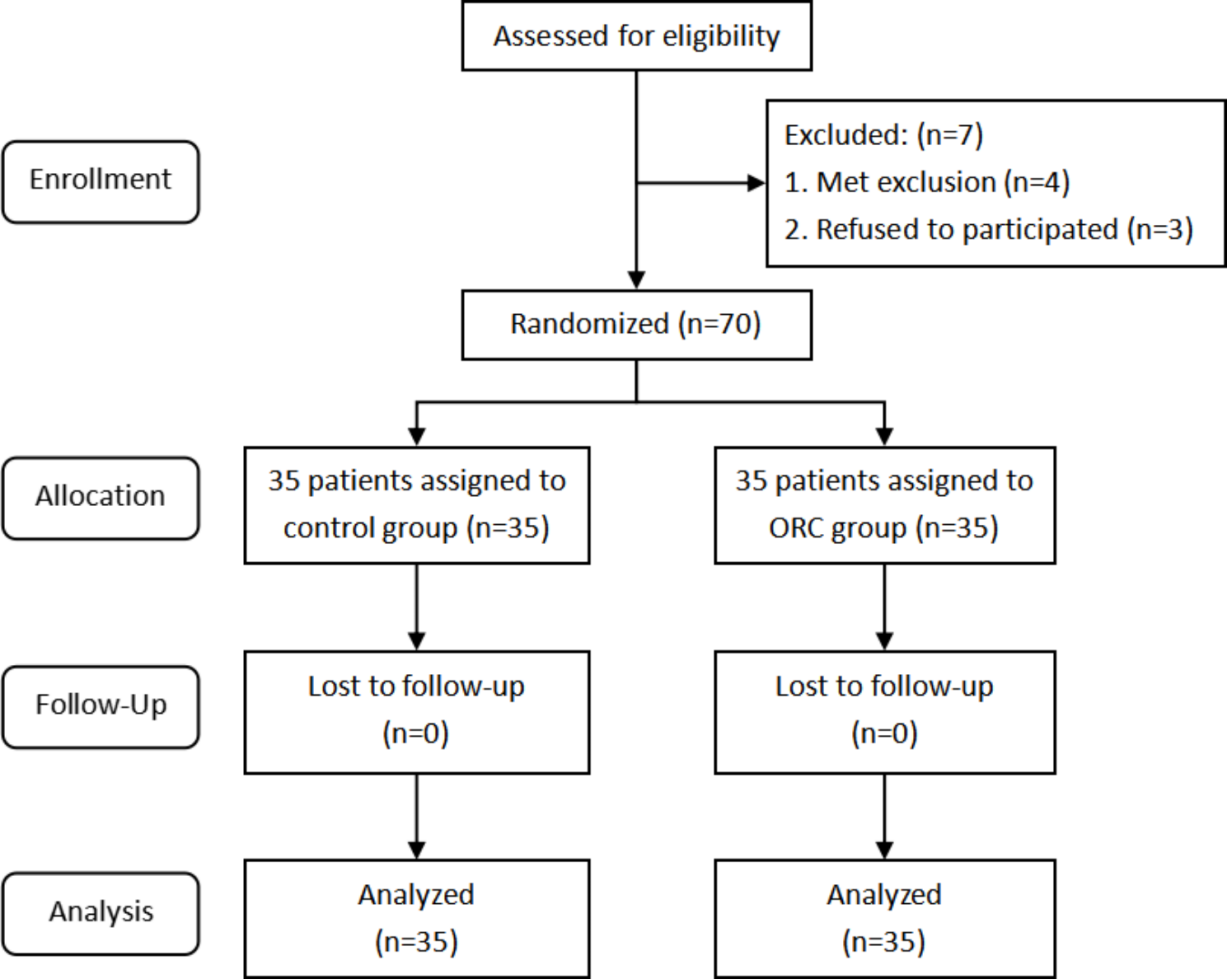
Flow chart of the patients included in the study

Seventy-seven patients scheduled for initial unilateral TKA participated in this trial, among which seven patients were excluded for the following reasons: four patients were ineligible according to the exclusion criteria and three patients declined to participate in this trial. The remaining 70 patients were randomly arranged into the control (n = 35) and ORC (n = 35) groups(Figure 2). All indicators of these patients during hospitalization were recorded, and a 6-week follow-up was performed after the patients were discharged to record complications.

The baseline data of the patients after admission were collected and analyzed. The results showed no statistical difference in the baseline data between the two groups, indicating that the two groups were comparable (Table 1). In addition, no statistical difference was found in the preoperative coagulation indicators between the two groups (Table 1).

**Table 1:** Demographic baseline information of patients

	Control Group (n = 35)	ORC Group (n = 35)	P value
Gender(male/female, n)	13/22	13/22	1
Age(year)	65.43 ± 5.86	64.86 ± 6.11	0.691
BMI(kg/m^2^)	25.23 ± 2.60	25.70 ± 2.50	0.445
ASA grade(II/III, n)	30/5	29/6	0.743
Preoperative-Hb(g/L)	133.26 ± 14.38	135.63 ± 15.19	0.505
Preoperative-Hct(%)	40.44 ± 3.88	41.16 ± 4.55	0.479
Platelet count(×10^9^/L)	207.03 ± 62.53	212.46 ± 55.80	0.703
PBV(ml)	4135.63 ± 547.33	4128.47 ± 553.29	0.957
Preoperative coagulation index			
PT(s)	11.37 ± 1.21	11.53 ± 0.92	0.791
PTA(%)	108.02 ± 11.67	107.97 ± 9.84	0.805
INR	0.94 ± 0.096	0.94 ± 0.073	0.814
APTT(s)	29.98 ± 2.71	29.88 ± 3.09	0.889
TT(s)	16.64 ± 0.85	16.71 ± 1.05	0.745
FIB(g/L)	3.05 ± 0.54	3.19 ± 0.69	0.340

BMI: body mass index. ASA grade: American Society of Anesthesiologists physical status classification. PBV: preoperative blood volume. PT: prothrombin time. PTA: prothrombin time activity. INR: international normalized ratio. APTT: activated partial prothrombin time. TT: thrombin time. FIB: fibrinogen

Total blood loss, Hb drop values, intraoperative blood loss, hidden blood loss, and operation time were statistically analyzed(Table 2). The total blood loss was 1052.25 ± 308.44 mL in the control group and 902.32 ± 307.82 mL in the ORC group, suggesting that the total blood loss in the ORC group was markedly lower than that in the control group (*P* < 0.05). The Hb drop value was 28.63 ± 11.19 mL in the ORC group, insignificantly lower than 32.29 ± 10.28 mL in the control group (*P* > 0.05). The hidden blood loss was 801.61 ± 298.80 mL in the ORC group, which was substantially lower than that in the control group (949.96 ± 297.59 mL) (*P* < 0.05). Also, the intraoperative blood loss in the ORC group (100.71 ± 14.51 mL) was insignificantly lower as compared to that in the control group (102.29 ± 14.47 mL) (*P* > 0.05). The same trend was observed for the operation time, as evidenced by an insignificantly shorter operation time in the ORC group (89.77 ± 13.46 min) than in the control group (91.4 ± 15.19 min) (*P* > 0.05).

**Table 2:** Comparison of perioperative data of patients between the two groups

	Control Group (n = 35)	ORC Group (n = 35)	P value
TBL(ml)	1052.25 ± 308.44	902.32 ± 307.82	0.046
Hb drop(g/L)	32.29 ± 10.28	28.63 ± 11.19	0.159
IBL(ml)	102.29 ± 14.47	100.71 ± 14.51	0.651
HBL(ml)	949.96 ± 297.59	801.61 ± 298.80	0.041
Operation time(min)	91.4 ± 15.19	89.77 ± 13.46	0.636

TBL: total blood loss. IBL: intraoperative blood loss. HBL: hidden blood loss

The coagulation indicators D-dimer and FDPs were tested before surgery and on 1 and 3 days after surgery, which demonstrated no statistical difference between the control and ORC groups (*P* > 0.05) (Table 3). The inflammatory indicators CRP and ESR were examined before surgery, on postoperative day 1, and on postoperative day 3. The results revealed no statistical difference between the two groups (*P* > 0.05) (Table 4).

**Table 3:** Comparison of coagulation indicators of patients between the two groups

		Control Group M(IQR)	ORC Group M(IQR)	P value
D-dimer(mg/L)	Preoperative	0.39(0.14)	0.43(0.20)	0.121
	Postoperative 1 day	2.72(3.50)	3.12(2.39)	0.626
	Postoperative 3 day	1.19(1.08)	0.92(0.79)	0.264
FDP(mg/L)	Preoperative	1.54(0.50)	1.70(1.00)	0.279
	Postoperative 1 day	8.20(10.83)	9.80(10.20)	0.626
	Postoperative 3 day	3.60(3.76)	3.52(1.76)	0.226

**Table 4:** Comparison of perioperative inflammatory indexes of patients between the two groups

		Control Group M(IQR)	ORC Group M(IQR)	P value
CRP(mg/L)	Preoperative	0.95(1.31)	1.17(2.04)	0.585
	Postoperative 1 day	19.94(20.19)	19.87(20.30)	0.879
	Postoperative 3 day	44.78(36.87)	35.86(34.68)	0.428
ESR(mm/H)	Preoperative	9.00(9.00)	10.00(6.00)	0.764
	Postoperative 1 day	11.00(10.00)	13.00(13.00)	0.219
	Postoperative 3 day	23.00(19.00)	24.00(17.00)	0.805

Postoperatively, patients in both groups were evaluated, and none of the patients received blood transfusion. During hospitalization and 6-week postoperative follow-up, 11 cases of intermuscular venous thrombosis and 5 cases of poor wound healing were observed in the control group, whereas 9 cases of intermuscular venous thrombosis and 4 cases of poor wound healing were found in the ORC group. No DVT, PE, or early infection was observed in either group(Table 5). In conclusion, the incidence of postoperative complications was not statistically different between the two groups (*P* > 0.05).

**Table 5:** Complications during the 6-week follow-up (n, %)

	Control Group(n = 35)	ORC Group(n = 35)	P value
Transfusion rate	0(0%)	0(0%)	
Deep vein thrombosis	0(0%)	0(0%)	
Intermuscular venous thrombosis	11(31.4%)	9(25.7%)	0.597
Pulmonary embolism	0(0%)	0(0%)	
Wound complications	5(14.3%)	4(11.4%)	1.0
Early infection	0(0%)	0(0%)	
Unplanned readmission	0(0%)	0(0%)	

## Discussion

In our study, the total blood loss was higher in the control group (1052.25 ± 308.44 mL) than in the ORC group (902.32 ± 307.82 mL), validating the prior result that the use of ORC could decrease blood loss in the field of surgery. This result can be explained by two facts: (i) after encountering blood in the joint cavity, ORC changes to dark brown or black gelatinous substances due to the degradation of red blood cells and subsequent production of acid hematin, which promotes clot formation; (ii) the use of ORC produces the local low-pH environment, promoting vasoconstriction and provides a scaffold for platelet adhesion and aggregation, which reduces blood loss.

Perioperative blood loss during TKA consists of two main parts, including intraoperative blood loss (IBL) and hidden blood loss (HBL)our data revealed that the intraoperative blood loss was 102.29 ± 14.47 mL in the control group, whilst intraoperative were 100.71 ± 14.51 mL in the ORC group. Intraoperative blood loss in the two groups was almost insignificant different, which might be due to the fact that the surgery was almost complete when ORC was placed such that ORC had little effect on reducing blood loss throughout the surgery.

In general, blood loss measured and recorded after TKA is only intraoperative blood loss and postoperative drainage, which, however, do not consist of the hidden blood loss caused by the combination of blood infiltration into tissues, residual blood in the knee, and damage to red blood cells from high levels of free fatty acids in the blood [13, 14].First, hidden blood loss is of clinical importance because it accounts for approximately 55% of total blood loss [15]. Second, hidden blood loss may be a cause of postoperative swelling and pain in the knee, which can affect the ability to perform early postoperative functional exercises and enhance the risk of postoperative infection [14]. Therefore, the reduction of hidden blood loss plays a great role in the rehabilitation exercise of patients. In this study, the control group had a hidden blood loss of 949.96 ± 297.59 mL, which was significantly higher than that in the ORC group 801.61 ± 298.80 mL. This result indicates that the use of ORC can reduce persistent bleeding in the knee joint after surgery and benefit effective hemostasis for at least 7 days because of its degradation cycle of 7–14 days in vivo.

The perioperative risks after arthroplasty cannot be ignored, among which lower extremity venous thrombosis is one of the common and high-risk complications [16, 17]. The results unraveled no statistical difference in D-dimer and FDP concentrations between the two groups from the preoperative period to postoperative day 3. Meanwhile, the postoperative occurrence of thrombosis was not statistically different between the two groups. These results indicate that the use of ORC does not affect the coagulation function of patients and does not increase the incidence of venous thrombosis in the lower extremities.

Extensive bone resection and soft tissue damage during TKA can trigger inflammation. In addition to the property of promoting hemostasis, ORC has the function of producing a low-pH environment to exert broad-spectrum antibacterial effects. Furthermore, in vitro experiments have reported that ORC has antibacterial activity against both Gram-positive and Gram-negative strains, including drug-resistant strains, which can diminish the incidence of infections [18].Our data unveiled that on postoperative days 1 and 3, CRP expression in the ORC group was insignificantly lower than that in the control group, accompanied by statistically insignificantly different ESR. In addition, there were no cases of infection by the end of the follow-up.

Despite its good biocompatibility and bioabsorbability, ORC has been occasionally reported to result in some adverse events. For example, ORC implantation in the surgical cavity may change surgical scars after breast-conserving surgery [19]. After surgery, residual ORC may interfere with the results of imaging tests and the diagnosis of physicians [20]. In the present study, there was no misdiagnosis with imaging due to residual ORC until the end of follow-up.

In addition, the use of ORC can diminish the use of an electrocoagulation knife for hemostasis before suturing and also decrease some side reactions and complications associated with electrocoagulation, such as tissue damage, burns, bleeding, and nerve damage [21]Furthermore, prior research displayed that ORC could carry antibiotics in the form of physical carriers to achieve greater antimicrobial capability [22, 23]. In summary, ORC has a high potential for development since it is extremely malleable and rich in carrying capacity.

Regenerated oxidized cellulose, as a degradable hemostatic material derived from plants, can effectively reduce the total blood loss and hidden blood loss of patients after total knee arthroplasty under the premise of ensuring safety, and avoid transfusion due to excessive blood loss. In daily clinical work, it can play a positive role in postoperative rehabilitation and functional exercise of patients. Compared with the more hemostatic drugs currently used clinically, ORC has been proved by research to reduce blood loss without increasing additional risks. Avoid the complications of chemicals such as tranexamic acid.Our study has several limitations. First, the sample size was small, and all patients were from the same study center. Second, the different degrees of osteotomy and soft tissue release during surgery can interfere with the amount of blood loss. The severity of KNEE OA in patients was not graded in this experiment, which might affect the results. Third, postoperative functional factors were not assessed, and the follow-up period was short. Hence, the effect of ORC on the long-term knee function of patients after surgery is unknown. Fourth, This study did not include the widely used tranexamic acid group in current clinical practice. In the future, a further comparison of the clinical efficacy and safety between ORC and tranexamic acid should be conducted, aiming to provide a safer and more effective hemostatic solution for patients with contraindications to tranexamic acid usage.

## Conclusions

In conclusion, our prospective RCT study proved that regenerated oxidized cellulose can be used safely in vivo and can effectively reduce postoperative blood loss in patients, which is a potential method for preventing blood loss after TKA.

## Data Availability

The datasets used and/or analysed during the current study are available from the corresponding author on reasonable request.

## List of Abbreviations

ORC: Oxidized regenerated cellulose
TKA: Total knee arthroplasty
KNEE OA: Knee osteoarthritis
Hb: Hemoglobin
RCT: Randomized control trial
DVT: Deep vein thrombosis
PE: Pulmonary embolism
BMI: Body mass index
ASA: American Society of Anesthesiologists
PT: Physical status classification, prothrombin time
PTA: Prothrombin time activity
INR: International normalized ratio
APTT: Activatedpartial prothrombin time
FIB: Thrombin time(TT), and fibrinogen
FDPs: Fibrin degradation products
ESR: Erythrocyte sedimentation rate
CRP: C-reactive protein
